# Negative appendectomy rate in patients diagnosed with acute appendicitis

**DOI:** 10.1186/s12893-022-01852-0

**Published:** 2022-11-22

**Authors:** Wongsakorn Chaochankit, Aeraungkoon Boocha, Srila Samphao

**Affiliations:** Department of Surgery, Faculty of Medicine, Prince of Songkla University, Hat Yai, Songkhla 90110 Thailand

**Keywords:** Appendicitis, False appendectomy, Negative appendectomy, Normal appendix

## Abstract

**Background:**

Acute appendicitis is the most common cause of acute lower abdominal pain leading patients to the emergency department. This study aims to find the negative appendectomy rate in patients diagnosed with acute appendicitis from 2015 to 2019.

**Methods:**

This study was a retrospective cohort study in the patients preoperatively diagnosed with acute appendicitis and underwent appendectomy from January 2015 to December 2019. Negative appendectomy is defined as the final pathologic results confirmed normal, congestion or peri-appendicitis.

**Results:**

The study population was 892 patients which was 54.3% female. The five-year negative appendectomy rate was 8.6% (n = 77) and 70% in female (n = 54). The factors associated with increasing the negative appendectomy rate were female (OR 2.23, P = 0.003), age ≤ 40 years old (OR 2.35, P = 0.003), and no history of diarrhea (OR 2.42, P = 0.017). Whereas the factors related to decline in the negative appendectomy rate were white blood cell count (WBC) $\ge$ 10,000 (OR 0.39, P = 0.016), neutrophil (N) $\ge$ 75% (OR 0.28, P < 0.001), and positive appendicitis from ultrasonography of abdomen (OR 0.04, P < 0.001) or computed tomography of abdomen (OR 0.07, P < 0.001).

**Conclusion:**

The negative appendectomy rate was less than 10% in this study. Female, age 40 ≤ years old and history of diarrhea were related to increase in negative appendectomy. The factors that related to decline in negative appendectomy were leukocytosis with cells shift to the left, positive acute appendicitis from abdominal ultrasonography and CT scan. However, to request the further imaging studies to diagnose patients with suspected acute appendicitis depends on the risk and benefit to each patient and the choice of investigation.

## Background

Acute appendicitis is the most common cause of acute lower abdominal pain leading patients to the emergency department. The incidence rate of acute appendicitis is about 5.7–50 patients per 100,000 persons among developed countries [1]. The life-time risk of acute appendicitis is 6–7% per person and is 8.6% in men and 6.7% in women [2, 3]. Nowadays, appendectomy in acute uncomplicated appendicitis is the acceptable standard treatment. The diagnosis of acute appendicitis is a challenge because it is mainly based on clinical conditions, physical examinations and basic laboratory investigations which may be approached with a high index of suspicion [4, 5]. From Sarıcı et al. showed white blood cell and neutrophil counts that are biomarkers of inflammation were lower in liver transplant patients; however, the C-reactive level and red blood cell distribution width, markers of severe appendicitis, were higher in the liver transplant patients [6]. However, appendectomy in patients preoperatively diagnosed acute appendicitis can turn out to be a negative appendectomy which means the appendix is normal from the final pathological report. In the past, the incidence of negative appendectomy ranged from 20–25%, but the selected patients who underwent further investigation, especially computed tomography (CT) of the abdomen, could significantly decrease the incidence rate to 5–10% [7–10]. Akbulut et al. [11] showed that determining appendix in the ultrasound was an independent risk factor for the diagnosis of acute appendicitis. In patients ≥ 50 years, rate determination of perforated appendicitis was significantly higher. Accurate acute appendicitis diagnoses are crucial. Any delay in diagnosis may result in appendiceal perforation (13–37%) with significant morbidity and mortality, although the latter depends on patient characteristics. On the other hand, non-definitive acute appendicitis diagnoses result in increased negative appendectomy rates (10–30%) [12]. Nevertheless, many developing countries including Thailand have some limitations for the further investigations due to financial problems, lack of medical facilities and cost effectiveness. Consequently, the incidence of negative appendectomy varies depending on the institute [13]. The disadvantage of negative appendectomy is not only wasted time and unnecessary hospital cost, but also the increased risk of patients’ developing anesthesia and post-operative complications from the operations [14–18]. So, this study aims to find the negative appendectomy rate in patients diagnosed with acute appendicitis from 2015 to 2019 and the factors related with negative appendectomy.

## Methods

### Study population

Data from patients who were preoperatively diagnosed with acute appendicitis, then underwent appendectomy in Songklanagarind hospital from January 2015 to December 2019, were retrospectively collected. The exclusion criteria consisted of patients younger than 15 years old (the minority of cases which refers to individuals younger than the age of 16, consent to participate must be obtained from their parents or legal guardians. We asked our ethics committee to approve and consent to participate section and clarify whether written informed consents to participate were obtained from the parents or legal guardians of any participant under the age of 16) [10], patients who underwent appendectomy for other reasons without preoperative diagnosis of acute appendicitis, patients who had no pathological reports, and patients who underwent interval appendectomy. The ethics committee of Songklanagarind University approved the protocol.

### Data gathering

The data record was divided into two parts. The first part was the baseline characteristics of the patients that consisted of age, gender, body measurement, characteristics of abdominal pain, diarrhea, urologic complaint, gynecological complaint, physical examination of abdomen, and basic laboratory investigations including in Alvarado clinical diagnosis score, urine pregnancy test from Songklanagarind hospital database. Alvarado score consisted of several features and had one or two scores. The features of Alvarado score were compounded with migratory of pain, anorexia, nausea, tenderness in right lower quadrant, rebound tenderness, elevated temperature (BT $\ge$ 37.5 °C), leukocytosis (white blood cell $\ge$ 10,000) and shift of white blood cell count to the left (Neutrophil $\ge$ 75%) [4]. The range of this score was separated into three groups: score 1–4, score 5–6 and score 7–10 [4]. The imaging study results (ultrasonography of abdomen and CT scan of abdomen) for investigating acute appendicitis patients were collected but not all the patients needed imaging performed. Patient who had Alvarado score 5–6 or for whom clinicians were unsure of the clinical diagnosis of acute appendicitis underwent imaging investigations. The second part was the operative details and pathologic reports that were also collected from the hospital database. Negative appendectomy was defined as post appendectomy final pathologic results when confirmed normal, congestion, peri-appendicitis, or tumor without inflammation.

### Statistical analysis

Categorical data was compared using Fisher’s exact test. Non-normal distributed data was compared using analysis of variance. Univariate analysis was used to evaluate the factors associated with negative appendectomy. The parameters that had P < 0.2 from the univariate analysis were selected for the multivariate logistic regression model with backward elimination [19]. Logistic regression was used to measure the relationship between dependent variables and one or more than independents variables with a Mann–Whitney U-test or Kruskal–Wallis test.

## Results

**Fig. 1:**
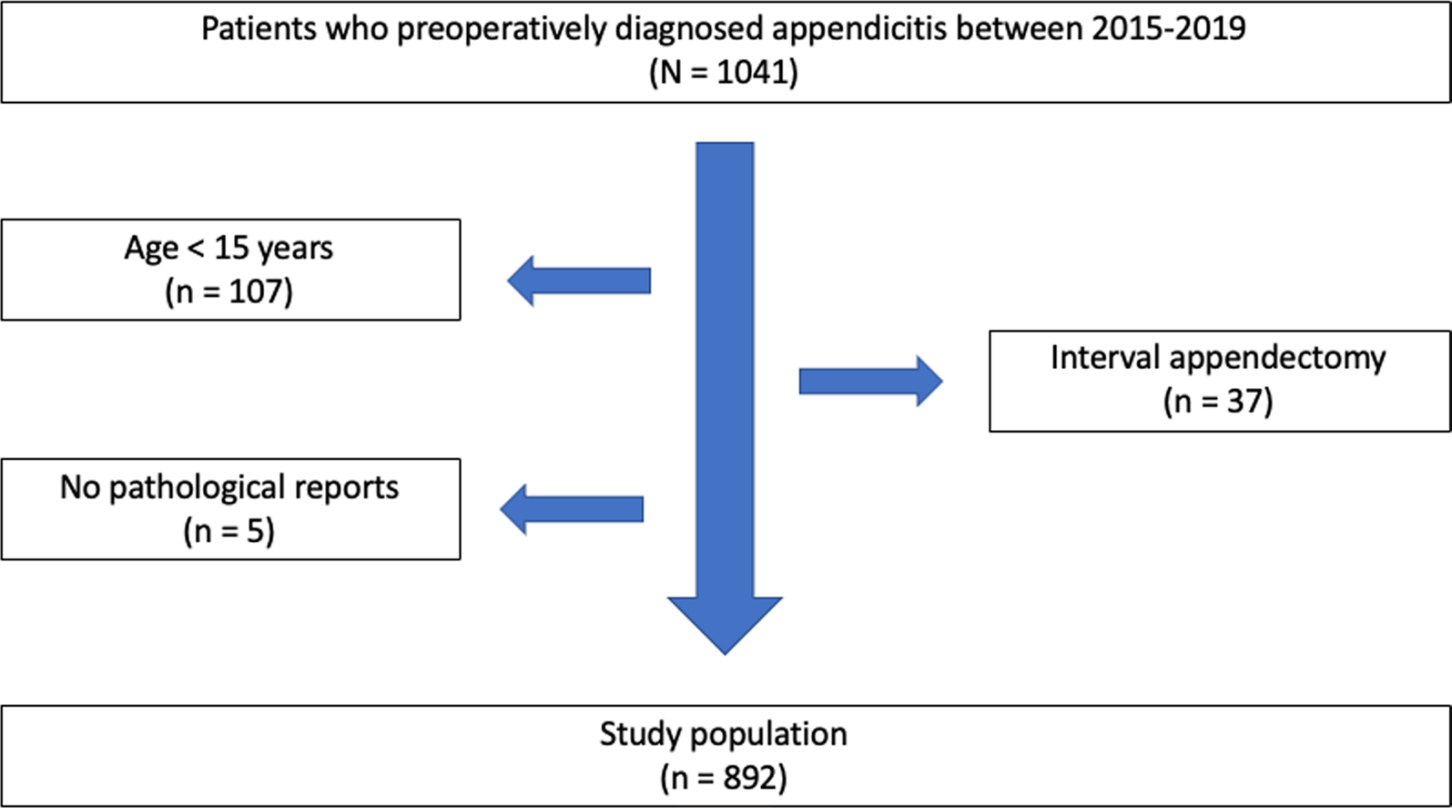
Flow of patient

Between January 2015 and December 2019, one thousand and forty-one patients were preoperatively diagnosed appendicitis. One hundred and ninety-seven patients were excluded from the study because one hundred and seven patients were younger than 15 years old, five patients had no final pathological reports, and thirty-seven patients underwent interval appendectomy. Therefore, eight hundred and ninety-two patients remained in this study as shown in Fig. 1.

The patients in this study were divided into two groups that consisted of negative appendectomy group and positive appendicitis group. Five-year negative appendectomy rate in patients diagnosed with acute appendicitis from 2015 to 2019 was 8.6% (n = 77) and the majority was female (70%). The median age was 31 years old in negative appendectomy group and 40 years old in positive appendicitis group with a significant difference in both groups. However, there was no significant difference in body mass index, clinical presentations including in Alvarado score, urologic complaint, gynecological complaint and urinalysis. The baseline characteristics are shown in Table 1.

**Table 1:** Baseline characteristics of patients who underwent appendectomy (N = 892)

Variables	Negative appendectomy (n = 77)	Positive appendectomy (n = 815)	P value
Age (year, IQR)	31 (21,43)	40 (24,57)	0.003
Female (n, %)	54 (70.1)	430 (52.8)	0.005
BMI (kg/m^2^, IQR)	22.2(19.5,25.4)	22.9 (20.8,25.7)	0.052
Pain localization (n, %)			0.436
RLQ area	29 (37.7)	254 (31.2)	
Periumbilical area	37 (48.1)	407 (49.9)	
Epigastric area	7 (9.1)	120 (14.7)	
Others	4 (5.2)	34 (4.2)	
Alvarado score (IQR)	7 (6,8)	7 (6,8)	0.004
Migratory of pain (n, %)	39 (50.6)	455 (55.8)	0.451
Anorexia (n, %)	37 (48.1)	327 (40.1)	0.218
Nausea (n, %)	40 (51.9)	491 (60.2)	0.195
RLQ tenderness (n, %)	74 (96.1)	792 (97.2)	0.485
Rebound tenderness (n, %)	34 (44.2)	438 (53.7)	0.136
Body temperature (°C, IQR)	37.5 (36.8,38)	37.2 (36.7,37.9)	0.312
White blood cell (UI, IQR)	12,370 (10,610,14,530)	14,770 (12,525,17,365)	< 0.001
Neutrophil (%, IQR)	77 (70, 82)	83 (77, 87)	< 0.001
Diarrhea (n, %)	14 (18.2)	79 (9.7)	0.033
Urologic complaint (n, %)	1 (1.3)	11 (1.3)	1.000
Gynecologic complaint (n, %)	3 (3.9)	7 (0.9)	0.048
Urinalysis (n, %)			0.071
Negative	71 (92.2)	775 (95.1)	
Positive	5 (6.5)	18 (2.2)	
Not perform	1 (1.3)	22 (2.7)	

All data are presented as median (IQR) unless indicated otherwise

Further imaging study (ultrasonography and CT scan of abdomen) was performed in almost half of all cases showing significant differences in both groups. However, the type of operation, operative time, length of hospital stays, and perioperative complications did not have any significant differences in either group as shown in Table 2.

**Table 2:** Imaging study, perioperative details of patients who underwent appendectomy (N = 892)

Variables	Negative appendectomy (n = 77)	Positive appendectomy (n = 815)	P value
Abdominal ultrasonography (n, %)			< 0.001
Negative	4 (5.2)	7 (0.9)	
Positive	10 (13)	243 (29.8)	
Equivocal	17 (22.1)	127 (15.6)	
Not perform	46 (59.7)	438 (53.7)	
Abdominal CT (n, %)			< 0.001
Negative	3 (3.9)	2 (0.2)	
Positive	18 (23.4)	291 (35.7)	
Equivocal	4 (5.2)	6 (0.7)	
Not perform	52 (67.5)	516 (63.3)	
Type of operation (n, %)			1.000
Open	69 (89.6)	734 (90.1)	
Laparoscopic	8 (10.4)	81 (9.9)	
Operative time (min, IQR)	110 (95,130)	115 (100,136)	0.644
LOS (days, IQR)	3 (2,4)	3 (2,4)	0.493
Intraoperative complication (n, %)	0 (0)	15 (1.8)	0.632
Postoperative complication (n, %)	5 (6.5)	43 (5.3)	0.598

All data are presented as median (IQR) unless indicated otherwise

Univariate analysis was used to evaluate the factors associated with negative appendectomy and showed that odds ratio (OR) of negative appendectomy was correlated with age ≤ 40 years (OR = 2.31, P = 0.001), female (OR = 2.1, P = 0.004), Alvarado score < 7 (OR = 1.88, P = 0.009), white blood cells (WBC) $\ge$ 10,000 (OR = 0.46, P = 0.013), neutrophil (N) $\ge$ 75% (OR = 0.32, P < 0.001), history of diarrhea (OR = 2.07, P = 0.022), gynecological complaint (OR = 4.68, P = 0.028), positive appendicitis from ultrasonography of abdomen (OR = 0.07, P < 0.001) and positive appendicitis from CT scan of abdomen (OR = 0.04, P < 0.001) as shown in Table 3.

**Table 3:** Univariate analysis between negative appendectomy and variables

Variables	OR (95% CI)	P value
Age ≤ 40 years old	2.31 (1.38, 3.85)	0.001
Female	2.10 (1.27, 3.49)	0.004
BMI > 23 kg/m^2^	0.72 (0.45, 1.15)	0.170
Pain localization pain: RLQ area (ref.)		
Periumbilicus	0.80 (0.48, 1.33)	0.382
Epigastrium	0.51 (0.22, 1.20)	0.123
Alvarado score < 7	1.88 (1.17, 3.02)	0.009
Migratory of pain	0.81 (0.51, 1.30)	0.383
Anorexia	1.38 (0.86, 2.21)	0.177
Nausea	0.71 (0.45, 1.14)	0.158
RLQ tenderness	0.72 (0.21, 2.44)	0.594
Rebound tenderness	0.68 (0.43, 1.09)	0.109
White blood cell $\ge$ 10,000	0.46 (0.25, 0.85)	0.013
Neutrophil $\ge$ 75%	0.32 (0.19, 0.52)	< 0.001
Body temperature $\ge$ 37.5 °C	1.43 (0.89, 2.28)	0.137
Diarrhea	2.07 (1.11, 3.86)	0.022
Urologic complaint	0.96 (0.12, 7.55)	0.970
Gynecologic complaint	4.68 (1.19, 18.48)	0.028
Positive UPT	1.14 (0.25, 5.29)	0.867
Positive Urinalysis	3.03 (1.09, 8.41)	0.033
Ultrasonography of abdomen: Positive	0.07 (0.02, 0.29)	< 0.001
Compute tomography of abdomen: Positive	0.04 (0.01, 0.26)	< 0.001
Non-retrocecal type	1.18 (0.71, 1.95)	0.533

Multivariate analysis logistic regression was performed with female, age ≤ 40 years old and history of diarrhea found to increase the double risk of negative appendectomy. The factors related to decrease the chance of negative appendectomy were positive appendicitis of abdominal ultrasonography and CT scan, white blood cells $\ge$ 10,000, and neutrophil $\ge$ 75%x as shown in Table 4.

**Table 4:** Determination of factors predicting negative appendectomy using backward stepwise logistic model

Variables	B	SE	Wald	Sig	OR (95% CI)
Age ≤ 40 years old	0.855	0.305	2.804	0.005	2.35 (1.29, 4.28)
Female	0.801	0.281	2.846	0.004	2.23 (1.28, 3.87)
Diarrhea	0.885	0.353	2.509	0.012	2.42 (1.21, 4.84)
Ultrasonography: positive	− 3.201	0.862	− 3.714	0.000	0.04 (0.01, 0.22)
Compute tomography: positive	− 2.703	1.085	− 2.491	0.012	0.07 (0.01, 0.56)
White blood cell $\ge$ 10,000	− 0.947	0.381	− 2.487	0.013	0.39 (0.18, 0.82)
Neutrophil $\ge$ 75%	− 1.269	0.288	− 4.405	0.000	0.28 (0.16, 0.49)
Constant	2.719	1.202	2.263	0.024	

## Discussion

This study showed negative appendectomy rate in patients diagnosed with acute appendicitis from 2015 to 2019, which was 8.6% (n = 77). When compared with other studies, the rate of negative appendectomy was 3–15%, which depended on the medical facilities and surgeons’ experience in each center. However, the negative appendectomy rate in our center was similar to the previous studies [20].

Furthermore, this study showed the relationships between several factors and negative appendectomy rate. The study found that female, age ≤ 40 years old and history of diarrhea were related to increase in the negative appendectomy rate. Whereas leukocytosis with cells shift to the left (WBC $\ge$ 10,000 and N $\ge$ 75%) and positive appendicitis from abdominal ultrasonography and CT scan were found to decrease negative appendectomy rate.

From this study, we found that female (70%) was more common in negative appendectomy group increasing negative appendectomy rate for 2.23 times when compared with male. Studies in the United States and Saudi Arabia also supported that the majority of negative appendectomy was found in female (65% and 64.3%, respectively). That could be explained by female patients having more chances of negative appendectomy due to gynecological problems mimicking acute appendicitis [13, 20–22].

Age was also a factor related to negative appendectomy. This study found that age less than forty significantly increased the rate of negative appendectomy 2.35 times when compared to age more than 40 years old (P = 0.003). According to the Courtney’s study [8], they found that more preoperative CT scan use in patients older than 45 years old did not significantly reduce the negative appendectomy rate. They assumed that it might have been a consequence of the initially low rates of negative appendectomy in this age group. Elderly patients needed to undergo further investigations, especially abdominal ultrasonography, or CT scan, than younger patients before receiving surgery because they had several differential diagnoses of abdominal pain when compared with younger age patients. So, the negative appendectomy rate in younger patients was higher than older patients as in our results.

History of diarrhea and abdominal pain might confuse the diagnosis eventually leading to negative appendectomy. These symptoms might mimic enterocolitis or irritable bowel syndrome (IBS). According to Lu’s study, Rome-II-defined IBS increased the rate of negative appendectomy (OR = 2.65, 95% CI 1.34–5.23) [23]. This reason might be explained by hyperperistalsis of the bowel movement and other patients’ abdominal pain complaints that might cause the clinicians’ misdiagnosis between acute appendicitis and other diseases of abdominal pain. These reasons might affect the patients undergoing appendectomy and occurrence of the negative appendectomy rate. Besides, the clinical features of acute appendicitis were less likely to have diarrhea. Therefore, if the patients have abdominal pain with diarrhea, the clinicians should be concerned when diagnosing acute appendicitis [23].

Complete blood count was the important tool to help the differential diagnosis in patients with suspected acute appendicitis. The leukocytosis was defined as WBC $\ge$ 10,000 and cells shift to the left was defined as neutrophil $\ge$ 75% [5]. These were two of the factors decreasing negative appendectomy. According to Muhammed Saaiq et al., using WBC cutoff level of 10,000/µL yielded the sensitivity of 92%. The negative appendectomy rates were decreased from 43.5% to 8.18% [24]. Another point from this study stated that the sensitivity, specificity, positive predictive value, and negative predictive value of elevated leukocyte counts were 91.81%, 43.55%, 81.77% and 65.85%, respectively [24].

Both abdominal ultrasonography and CT scan showed positive acute appendicitis that decreased the negative appendectomy rate in this study. Several large database studies, meta-analyses and single institution studies credited abdominal CT scan with reducing the negative appendectomy and in the landmark study of Rao et al., the CT rates in the United States had risen rapidly [25] and negative appendectomy rate of 1–3% had been reported [26, 27]. This study demonstrates that abdominal CT scan is the standard for diagnosing patients with suspected acute appendicitis. Moreover, many previous studies showed that imaging studies significantly impacted the decreasing incidence of negative appendectomy. According to Mariadason et al., CT scan use was beneficial in lowering the negative appendectomy rate from 9.2 to 3% [9]. However, our study had just 46% and 37% of patients who underwent abdominal ultrasound and abdominal CT scan, respectively. In developing countries including Thailand, the usage of further investigations especially CT scan of abdomen to diagnose acute appendicitis should be requested carefully due to the cost and availability of facilities in each center. Moreover, the negative appendectomy rate was quite low (11.1%) in patients who did not undergo any imaging studies in our study.

This study was a five-year retrospective single center study. The data was collected in a high-volume medical university center. The limitation of this study was the incomplete history taking of comorbidity or other illness which could be analyzed more precisely, and the incomplete data of imaging details compared with final pathologic results which could imply sensitivity and specificity. A prospective study collecting patient and investigation data in more details might be helpful to clarify the risk, predictive factor of negative appendectomy and might show the accuracy and precision of our institutes’ facilities.

## Conclusion

The negative appendectomy rate was less than 10% in this study. Female, younger patients (age ≤ 40 years) and history of diarrhea should be of high concern because they may increase the negative appendectomy rate. The other factors that decreased the negative appendectomy rate were leukocytosis with cells shift to the left and positive acute appendicitis from ultrasonography or computed tomography of abdomen. However, to request the further imaging studies to diagnose patients with suspected acute appendicitis depends on the risk and benefit to each patient and investigation.

## Data Availability

The datasets used and/or analysed during the current study are available from the corresponding author on reasonable request.

## Abbreviations

IQR: Inter quartile range
kg: Kilogram
m: Meter
RLQ: Right lower quadrant
CT: Computed tomography
min: Minute
LOS: Length of stay
OR: Odd ratio
CI: Confidence interval
kg: Kilogram
m: Meter
RLQ: Right lower quadrant
ref: Reference
UPT: Urine pregnant test
